# The Minus Approach Can Redefine the Standard of Practice of Drinking Water Treatment

**DOI:** 10.1021/acs.est.2c09389

**Published:** 2023-04-19

**Authors:** Elliot Reid, Thomas Igou, Yangying Zhao, John Crittenden, Ching-Hua Huang, Paul Westerhoff, Bruce Rittmann, Jörg E. Drewes, Yongsheng Chen

**Affiliations:** † School of Civil and Environmental Engineering, Georgia Institute of Technology, Atlanta, Georgia 30332, United States; ‡ Brook Byers Institute for Sustainable Systems, Georgia Institute of Technology, Atlanta, Georgia 30332, United States; § Nanosystems Engineering Research Center for Nanotechnology-Enabled Water Treatment, School of Sustainable Engineering and The Built Environment, Ira A. Fulton Schools of Engineering, Arizona State University, Tempe, Arizona 85287, United States; ∥ Biodesign Swette Center for Environmental Biotechnology, Arizona State University, Tempe, Arizona 85287, United States; ⊥ Chair of Urban Water Systems Engineering, Technical University of Munich, 85748 Garching, Germany

**Keywords:** drinking water treatment, membranes, biofiltration, artificial intelligence, sustainability

## Abstract

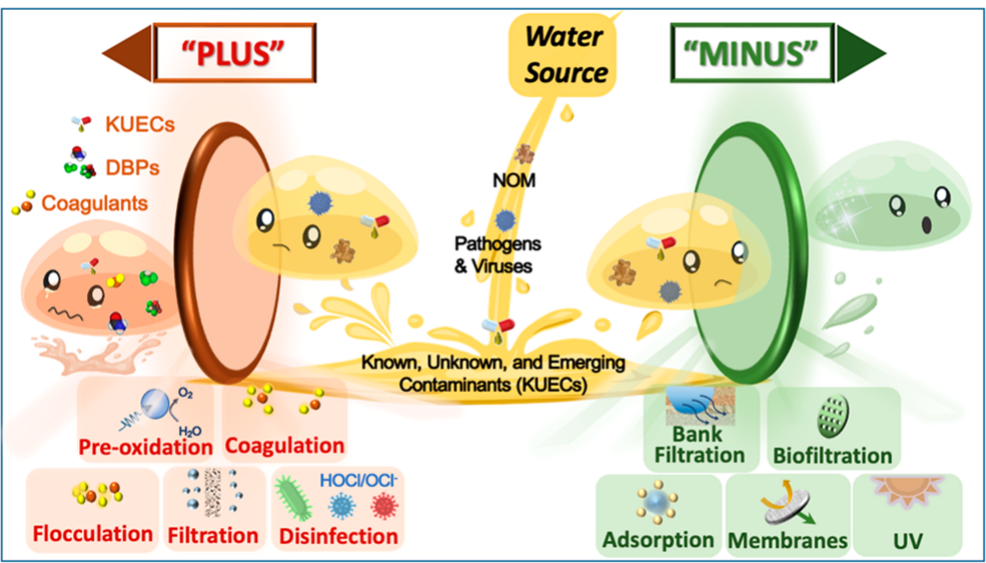

Chlorine-based disinfection for drinking water treatment (DWT) was one of the 20th century’s great public health achievements, as it substantially reduced the risk of acute microbial waterborne disease. However, today’s chlorinated drinking water is not unambiguously safe; trace levels of regulated and unregulated disinfection byproducts (DBPs), and other known, unknown, and emerging contaminants (KUECs), present chronic risks that make them essential removal targets. Because conventional chemical-based DWT processes do little to remove DBPs or KUECs, alternative approaches are needed to minimize risks by removing DBP precursors and KUECs that are ubiquitous in water supplies. We present the “Minus Approach” as a toolbox of practices and technologies to mitigate KUECs and DBPs without compromising microbiological safety. The Minus Approach reduces problem-causing chemical addition treatment (i.e., the conventional “Plus Approach”) by producing biologically stable water containing pathogens at levels having negligible human health risk and substantially lower concentrations of KUECs and DBPs. Aside from ozonation, the Minus Approach avoids primary chemical-based coagulants, disinfectants, and advanced oxidation processes. The Minus Approach focuses on bank filtration, biofiltration, adsorption, and membranes to biologically and physically remove DBP precursors, KUECs, and pathogens; consequently, water purveyors can use ultraviolet light at key locations in conjunction with smaller dosages of secondary chemical disinfectants to minimize microbial regrowth in distribution systems. We describe how the Minus Approach contrasts with the conventional Plus Approach, integrates with artificial intelligence, and can ultimately improve the sustainability performance of water treatment. Finally, we consider barriers to adoption of the Minus Approach.

## Abstract 2

The Minus Approach is a toolbox of practices and technologies to enhance drinking water quality by minimizing uncertain microbial and chemical risks.

## 1 A Global Cause for Concern

### 1.1 Disinfection Byproducts (DBPs) and Known, Unknown, and Emerging Contaminants (KUECs) in Drinking Water

Microbial pathogens in drinking water can cause acute diseases that dramatically increase morbidity and mortality risks. Because it is imperative that the product water’s total pathogen concentrations meet an acceptable level for a very low risk of infectivity, improvements in sanitation, filtration, and disinfection in the 20th century significantly reduced the threat of acute disease from drinking water.^1^ Ultraviolet (UV) light and chemical disinfectants inactivate microbes, and they are applied before, during, and after drinking water treatment (DWT) to ensure plant-to-tap microbiological safety. Primary disinfectants inactivate pathogens, while secondary disinfectants minimize microbial regrowth or harm from the inadvertent intrusion of microbes into drinking water distribution systems (DWDS) (e.g., infiltration and pipe failures).

Water sources are at risk of contamination from thousands of legacy, current, and newly synthesized chemicals and their transformation byproducts; they occur in drinking water supplies or are unintentionally formed during addition of chemical disinfectants at DWT plants (DWTPs). In 2014, the American Chemical Society (ACS) registered 89 million inorganic and organic compounds and 65 million gene sequences,^2^ and their environmental discharge creates complex, poorly characterized water matrices. Known, unknown, and emerging contaminants (KUECs) is an umbrella term for constituents not routinely monitored; they often are present in ultralow concentrations (e.g., parts per trillion or parts per billion). KUECs include but are not limited to pharmaceuticals and personal care products (PPCPs), micro- and nanoplastics, endocrine-disrupting compounds (EDCs), surfactants, plasticizers, pesticides, fertilizers, short- and long-chain per- and polyfluoroalkyl substances (PFAS), harmful microbial products (e.g., algal/cyanobacterial toxins), radioactive isotopes, and antibiotic-resistance genes.^3,4^

The disinfectants that inactivate microbes also react with dissolved precursors in water, including natural (NOM) or effluent (EfOM) organic matter, organic contaminants (e.g., KUECs), nitrogenous compounds, and halides (e.g., bromide and iodide), to form cyto- and genotoxic disinfection byproducts (DBPs) during treatment.^5,6^ DBPs regulated by state or federal law in the United States and the European Union (EU) include trihalomethanes (THMs), haloacetic acids, nitrosamines, bromate, chlorate, and chlorite at either nanogram, microgram, or milligrams per liter concentrations, depending on the DBP. However, many unregulated DBPs have been reported, including more than 700 unique DBPs detected in drinking water as of 2017,^3^ and the formation pathways, human metabolism, and toxicity mechanisms of many of these compounds remain ill-defined. Additionally, characterized DBPs account for only 30% of the total organic halogenated (TOX) composition in chlorinated waters on a median basis, suggesting that a myriad of other unidentified DBPs are present.^7^

Multiple disinfectants without free chlorine (HOCl/OCl^–^) were introduced, including chlorine dioxide, chloramines, and ozone, because (1) some important pathogens (e.g., *Giardia* and *Cryptosporidium*) are more resistant to chlorine than most pathogens^8^ and (2) water purveyors wanted to minimize chlorinated DBPs. Unfortunately, these disinfectants form their own suites of DBPs, many of which are uncharacterized and continue to be detected following advancements in analytical chemistry.^9^ Often, controlling one class of DBPs can exacerbate the risk of forming another class of DBPs that may be more toxic; e.g., switching from free chlorine to chloramines decreases the level of THM formation but increases the level of *N*-nitrosodimethylamine (NDMA) and *N*-nitrosodiethylamine (NDEA) formation.^10,11^ Regardless of the chemical disinfectant, DBP formation is sensitive to and varies temporally and spatially on the basis of source water composition, temperature, pH, and disinfection contact time,^12^ making it difficult to predict DBP composition and risk. Overall, chemical-based disinfection creates a trade-off between acute disease (e.g., cryptosporidiosis) and increased risks of chronic disease associated with long-term DBP exposure (e.g., bladder cancer).^13^

### 1.2 Increasing Levels of KUECs in Source Waters

Population growth and other increasing discharges to surface waters are reshaping watersheds once considered pristine sources of drinking water. Climate change creates additional challenges (higher average water temperatures, heavier rains, more frequent fires, and increased instances of flooding and extended droughts) that will exacerbate adverse water quality events (e.g., heightened pathogen survival and mobility, altered NOM composition, increased agricultural runoff, and eutrophication).^14−17^

**Figure 1:**
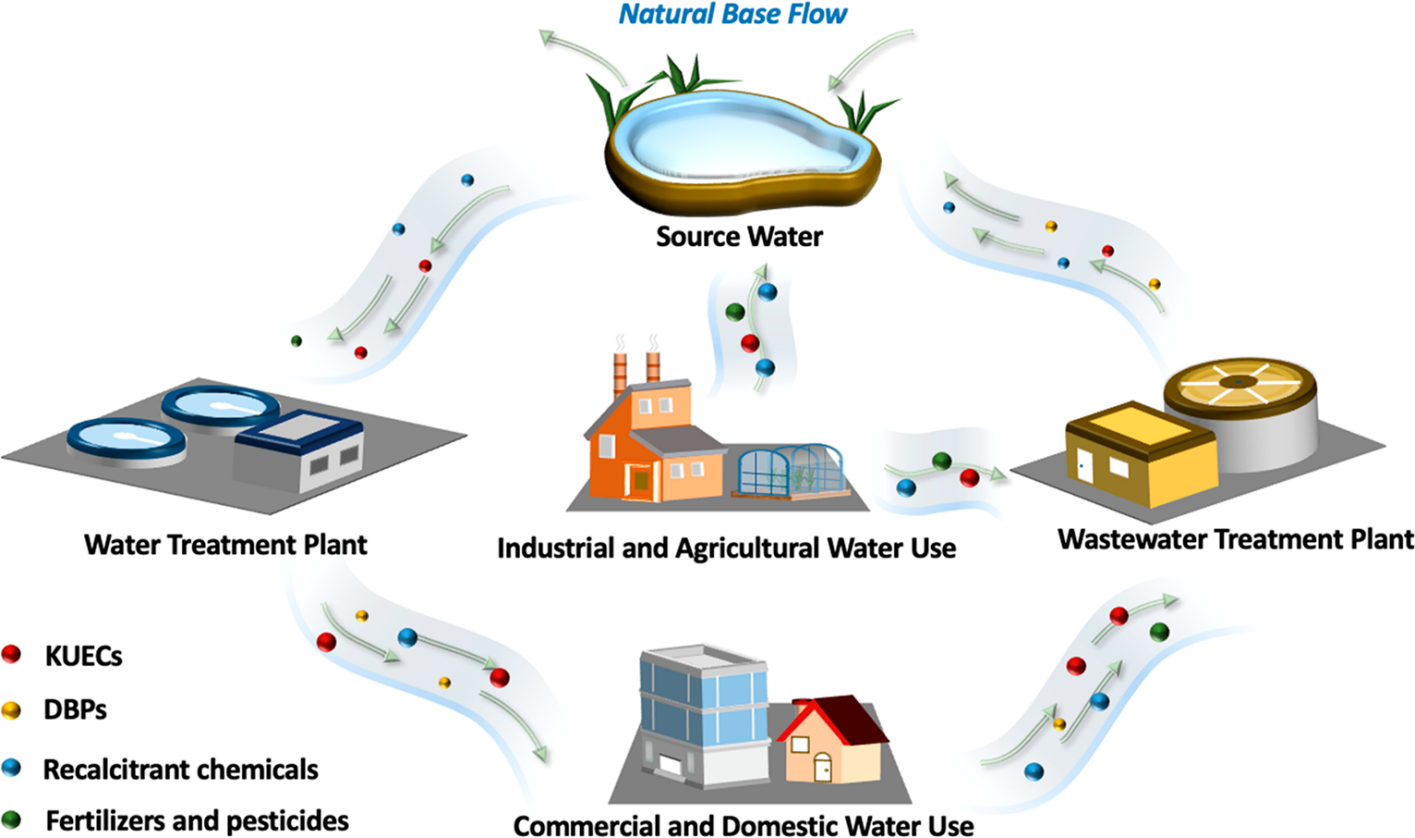
Increasingly complex and interconnected water cycle that increases the potential risks of KUECs and DBPs in drinking water.

Figure 1 illustrates the increasingly interconnected water cycle and emphasizes *de facto* reuse, the unplanned and unintended reuse of partially treated wastewater.^18,19^ Wastewater treatment plants (WWTPs) receive sewage, stormwater, agricultural runoff, and sometimes industrial wastewaters; incomplete KUEC removals at WWTPs further pollute natural water resources during effluent discharge.^20^ In these waterways, physical, chemical, and biological transformations can occur, but many recalcitrant organic contaminants (e.g., PFAS) and ones with favorable properties for mobility (e.g., high-polarity, low-molecular weight, high-solubility compounds),^21^ like 1,4-dioxane, do not readily degrade. Thus, as waterways are tapped for DWT, the numerous, disperse, and poorly regulated pollutant sources create risks due to the presence of DBP precursors, DBPs, and KUECs. These risks are readily apparent in rivers where upstream WWTPs discharge into rivers with lower mean seasonal stream flows (i.e., less natural dilution increases the level of *de facto* reuse at downstream DWTPs).^18,19,22,23^ A prime example of *de facto* reuse is the Trinity River in Texas, a major source of water for the Houston metropolitan area, which can be almost entirely comprised of wastewater effluents under base-flow conditions.^24,25^

### 1.3 Public Health and Environmental Impacts

DBPs and KUECs fall into three risk categories: (1) known compounds with known toxicity, (2) known compounds with unknown toxicity, and (3) unknown compounds that have not yet been detected and have unknown toxicity. Some DBPs present potential threats to human health as carcinogens, mutagens, and teratogens,^26^ but due to the hundreds to thousands of DBPs formed during disinfection and the large uncertainty of DBP composition, determining the true etiological chemical agents of disease is immensely challenging. Although THMs were once thought to be the drivers for an increased risk of bladder cancer, recent toxicology studies show that nitrosamines, halonitriles, and other non-nitrogenous DBPs may be more responsible for this risk.^27,28^ More holistic strategies for controlling DBPs instead of identifying, regulating, and controlling one DBP class at a time are needed.

Some KUECs, such as pharmaceuticals that are designed to be metabolized by human biochemical pathways, exist at low concentrations and currently lack sufficient evidence to suggest that they threaten human health.^29^ However, most studies have examined acute toxicities, and research investigating the synergistic (or antagonistic) effects of multiple pharmaceuticals at environmentally relevant concentrations on chronic toxicity is lacking.^29^ Additionally, evidence is mounting that continuous exposure to some pharmaceuticals in the natural environment can be detrimental to aquatic life (e.g., mixtures of antidepressants can alter piscine circadian rhythm),^30^ and pharmaceuticals, like many other KUECs, can react with various disinfectants to form new DBPs.^31^ Some other KUECs present in drinking water, such as PFAS and 1,4-dioxane, have detrimental human health effects.^32,33^ The human health and environmental effects of long-term exposure to KUECs and DBPs at very low concentrations in drinking water are not fully known. Despite the uncertainty, the potential risks that KUECs and DBPs present should be addressed with precautionary principles.^34^

## 2 Lessons Learned from Around the Globe

The multifaceted but poorly understood risks associated with DBPs have inspired several European countries, in particular, The Netherlands, Germany, Austria, and Switzerland, to abandon or significantly reduce primary and secondary disinfection in DWT. The Netherlands has achieved this without increased incidence of waterborne disease by (1) tapping the best source water available, (2) using multibarrier biological and physical treatment technologies (including ozone when absolutely necessary to meet potable standards), (3) providing biologically stable water distributed through biostable materials, (4) preventing downstream contamination during distribution (e.g., shortening retention times and avoiding stagnant water), and (5) routinely monitoring and correcting failures during treatment and distribution (e.g., maintaining sufficient pressure in the DWDS to prevent infiltration).^35,36^ Despite the near elimination of disinfectants in DWT, Dutch and German finished waters consistently do not contain microbial pathogens at a level of health concern.^35,36^

One strategy that has greatly reduced dependence on chemical disinfection is removal of biodegradable organic matter (BOM) via creation of biologically stable water that reduces the residual disinfectant dose and DBP formation potential.^37^ The most widely used parameter for measuring biostability is assimilable organic carbon (AOC), which is the most rapidly biodegradable portion of NOM (e.g., ketones and carboxylic acids). AOC closely aligns with the potential for bacterial growth during distribution.^38^ AOC concentrations that stimulate excessive growth range from 10 to 100 μg of C/L, depending on the concentration of disinfectant residual in finished water,^39^ and The Netherlands uses a biological stability guideline of <10 μg of C/L for its unchlorinated water.^40^ Ensuring acceptably low AOC concentrations in finished water is especially important for large DWDS that deliver water from centralized DWTPs, as it lessens biofilm growth and the opportunity for pathogenic microorganisms to contaminate potable water.^41^ The experiences in these European nations clearly demonstrate that biologically stable drinking water can be produced and safely distributed to consumers.

## 3 Reducing Uncertainties in Drinking Water Using the “Minus Approach”

### 3.1 Philosophy of the Minus Approach

While the conventional “Plus Approach” for DWT has mostly mitigated acute, waterborne microbial disease, it amplifies and is poorly equipped to manage the chemical risks posed by KUECs, DBPs, and DBP precursors. Thus, we advocate a “Minus Approach” that is founded on the knowledge that the removal of pathogens, traditional water contaminants, KUECs, and DBP precursors from raw water can be more simply and safely achieved by multibarrier physical and biological treatments. The Minus Approach can provide safe, biologically stable drinking water and ensures less human health risk by minimizing the level of DBPs produced by disinfection compared to the Plus Approach. It is important to realize that the Minus Approach can concentrate and remove many KUECs from drinking water; however, more research is needed to develop technologies to effectively decompose them. In addition, we need to develop and use alternative treatment chemicals with minimal downstream consequences (i.e., green chemicals).^42^

The graphical abstract compares the Minus Approach to the conventional Plus Approach. The Plus Approach treats surface water via preoxidation, coagulation, flocculation, filtration, and disinfection to remove NOM, pathogens, and inorganic contaminants, in turn producing a potable product with improved taste, odor, turbidity, and overall quality. However, finished water contains DBPs and KUECs that pose uncertain health risks for consumers. Relying on bank filtration, biofiltration, membranes, and UV light, the Minus Approach produces drinking water of equivalent or better quality (acceptable pathogen concentrations and minimized contamination by DBPs and KUECs).

Below, we discuss technologies, materials, and water practices that embody the Minus Approach, a toolbox of options for safer drinking water. We begin by emphasizing that the Minus Approach encourages source protections to provide higher-quality source water. Then, we describe technologies that can provide potable water without large chemical additions in mainline treatment and discuss the strategy for distributing water without increasing risks. Finally, we discuss residual management strategies, sustainability and economics, how to make water treatment systems more robust through machine learning (ML) and artificial intelligence (AI), and potential impediments to implementing the Minus Approach.

### 3.2 Protecting Ever-Changing Source Water

When possible, maintaining a pristine water source is paramount to providing the highest-quality water. New York City’s (NYC) tap water has been called “the champagne of drinking water in the United States”, and their drinking water source network is the largest supply of unfiltered drinking water in the world, resulting in significant cost savings.^43^ In 1997, multiple stakeholders signed the New York Cicty Watershed Memorandum of Agreement, which established protections for the Catskill, Delaware, and Croton watersheds in a variety of ways such as drafting new rules and regulations (e.g., more stringent pesticide concentration limits in runoff), acquiring land to establish riparian protection zones, and performing routine maintenance and monitoring of decentralized wastewater treatment systems (e.g., septic tanks) in the watersheds.^43^ New York City remains the largest utility in the United States to use nonfiltered water supplies in part due to control over population growth and activities in the watershed. Filtration-associated cost savings allow for the employment of advanced DWT technologies. For instance, the Catskill-Delaware UV-DWTP, which supplies 90% of New York City’s drinking water, uses UV disinfection to ensure inactivation of *Cryptosporidium* and other pathogens, allowing for small doses of chlorine in finished water to comply with laws mandating residual disinfectant concentrations.

Although New York City’s case is unique, watershed protection does not have to be elaborate; simple safeguards like protected reservoirs can function as natural clarifiers that contain fewer contaminants (e.g., particles and *Cryptosporidium* oocysts),^44^ resulting in easier and less costly treatment. Moreover, pollution controls taken at upstream WWTPs can further decrease needed DWT by reducing chemical and pathogenic threats.^20,45^ The European Commission, as part of their Green Deal Policy, is emphasizing zero discharge of persistent chemicals.^46^ This approach includes equipping WWTPs (with capacities of ≥100 000 population equivalents) with advanced treatment, which can reduce the overall toxic load of micropollutants entering EU freshwater ecosystems by ∼40%.^47,48^

### 3.3 Minus Approach Technologies

#### 3.3.1 Bank Filtration and Managed Aquifer Recharge

**Figure 2:**
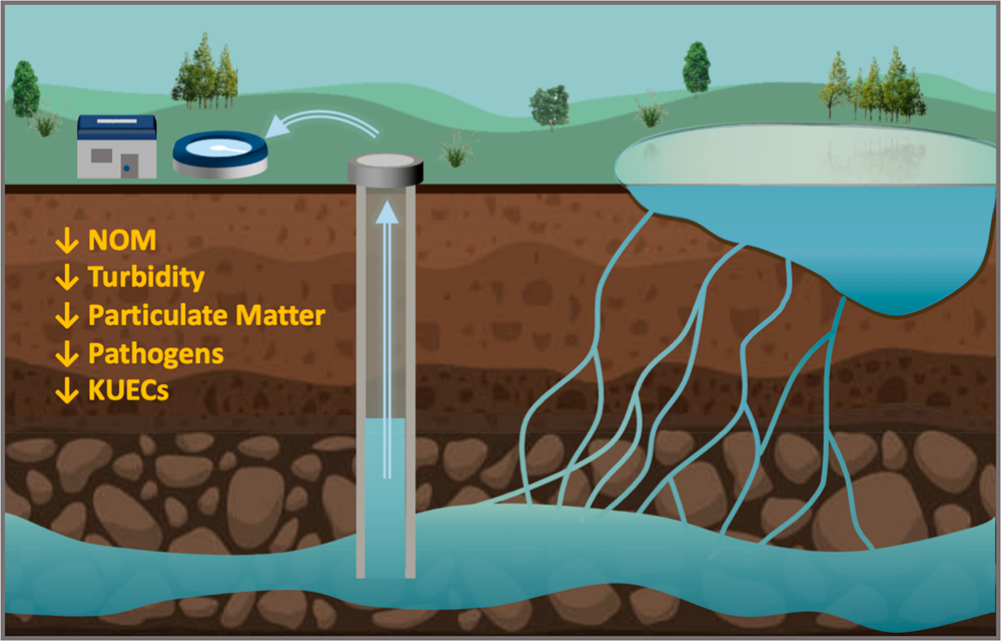
Lake-bank filtration is a multiobjective primary treatment process.

Most water purveyors cannot find new sources or maintain pristine sources like New York City. In those cases, the best practice is to adopt alternative multiobjective primary treatment processes. Two excellent examples are riverbank filtration (RBF) and lake-bank filtration (LBF), which are widely used in Europe and in some parts of the United States.^49^ LBF, depicted in Figure 2, and RBF are passive, sustainable means of natural treatment that take advantage of geochemistry, biology, and hydrology to reduce levels of NOM, turbidity, particulate matter, organic contaminants, and pathogens by tapping water from aquifers that are hydraulically connected to rivers. As water from a river or a lake is drawn into surrounding aquifers, it undergoes quality-improving processes: particle and pathogen filtration, ion exchange, biotransformation, and adsorption of organic constituents.^50,51^ These processes can also reduce DBP formation potential and remove many KUECs, including pharmaceuticals, pesticides, and EDCs.^51−53^

Because LBF and RBF are constrained by geology and hydrology, managed aquifer recharge (MAR) systems are engineered systems that pump source water into adjacent constructed infiltration/recharge basins and provide similar water-quality benefits.^54^ MAR performance can be enhanced through preozonation to improve biostability and post-treatment for the removal of iron and manganese, because these ions can be mobilized during subsurface passage.^55,56^ Manipulating redox conditions and manipulating substrate availability are additional tools; field-scale studies in Berlin, Germany, and Colorado, United States, demonstrated that sequential MAR systems employing both oxic and carbon-limited conditions enhanced the biotransformation of several KUECs compared to a conventional MAR system.^57,58^ These primary treatment processes allow countries like The Netherlands and Germany to forego secondary disinfection due to resultant biostability and to produce high-quality drinking water despite treating impaired source waters (e.g., the Rhine River and Elbe River) that receive municipal and industrial wastewater discharges.^19^

#### 3.3.2 Biofiltration

A core feature of the Minus Approach is biofiltration (BF) to make water biologically stable.^59^ Contaminants that support the growth of bacteria [e.g., BOM or inorganic electron donors like ammonium, ferrous iron, manganese(II), and sulfide] cause water to be biologically unstable. When such water is distributed, bacterial growth in the DWDS can lead to the deterioration of water quality, including increases in turbidity, tastes, odors, and corrosion, as well as loss of disinfectant residual and dissolved oxygen.

BF involves contacting biologically unstable water with a microbial biofilm growing on a porous medium. The medium most commonly employed is a packed bed that is intermittently backwashed, but it can also exist as a dedicated biofilm reactor (e.g., fluidized beds) when the feedwater contains too much BOM or particulate matter that would quickly clog a packed bed.^60^ The media can be chemically inert (e.g., sand in a slow-sand filter) or an adsorbent [e.g., granular activated carbon (GAC)], which over time begins to support an active biofilm in its macropores. GAC biofilters operate like dual-medium rapid filters, but with little or no chlorination of the bed. Slow-sand filters generally are a cheaper alternative if ample land area is available. Key factors that influence BF performance include the concentration of dissolved oxygen, nutrients (nitrogen and phosphorus), water temperature, empty bed contact time, medium size, hydraulic loading rate, and backwashing frequency and intensity.^61^

The Minus Approach can include ozonation prior to BF, as ozonation converts the less biodegradable fractions of NOM into BOM, which can also help remove many KUECs of concern in raw water (e.g., PCPPs and pesticides).^62,63^ Ozonation should be used cautiously when water contains relatively high bromide concentrations (to avoid bromate formation), and chlorination should not directly follow ozonation, as this can spur halonitromethane formation.^64^ Even in the absence of ozonation, BF can remove many KUECs and DBP precursors, particularly many PPCPs, low-molecular weight organics, N-DBP precursors, and pesticides,^65^ although removal of less biodegradable compounds may be minimal or require long contact times, especially at low temperatures.^66^ BF has proven to be a reliable process even in cold areas like Canada, where cold water temperatures were once thought to limit bacterial growth and feasibility to remove AOC.^67^ Although some parent KUECs may be “removed” by adsorption or biotransformation, they may not be completely mineralized but form transformation products that may be harder to remove because they are more hydrophilic than the parent compound.^68−70^

Although BF technologies are mature, contemporary biomolecular methods (i.e., metagenomics, transcriptomics, and proteomics) open opportunities to improve the monitoring, understanding, and optimizing of BF performance.^71^ The core principle of environmental biotechnology is managing microbial communities to provide services to society (e.g., detoxifying contaminants in water).^72^ For instance, amplicon sequencing and qPCR evidenced the increased abundance of key nitrifiers upon stimulation with a small dose of copper (<1 μg/L) in BF that previously had poor nitrification when treating groundwater.^73^

#### 3.3.3 Membranes

Pressure-driven membranes rely on size exclusion, cake formation, adsorption, and/or differential molecular diffusion to remove particles and solutes, and they avoid the inadvertent creation of new contaminants *in situ*.

Technologies applicable to DWT include microfiltration (MF), ultrafiltration (UF), nanofiltration (NF), and reverse osmosis (RO). MF and UF can remove particulate matter, some higher-molecular weight organics, and particles as small as viruses. NF can remove multivalent ions, while RO can remove nearly all ions; both can remove many mid-high-molecular weight KUECs (>200 Da) and a large portion of dissolved organic matter.^74,75^ Thus, these technologies can offer the particle removal benefits of conventional coagulation, flocculation, and filtration and provide disinfection and removal of dissolved compounds. In addition, membrane effluent quality is much more resilient to changes in influent water quality (e.g., storm-event turbidity changes).

Perhaps the greatest advantage of using NF and RO instead of conventional filtration processes is their enhanced ability to remove KUECs. High retention efficiencies of KUECs having a range of physicochemical properties can be attributed to size exclusion, electrostatic repulsion, and hydrophobic interactions, although these mechanisms are influenced by different operating conditions (e.g., pH, conductivity, and water source).^74,76^ Notably, many long-chain and short-chain PFAS can be removed through high-pressure membranes.^77^ NF is a superior choice for groundwater and surface water treatment due to its lower energy requirement and superior selectivity compared to those of RO.^78^ A great deal of research concerns the creation of “fit-for-purpose” NF membranes,^79^ which are modified structurally and/or chemically to provide precise solute separation at the subnanometer or subangstrom scale (e.g., the passage of Ca^2+^ ions to minimize remineralization needs and scaling propensity while retaining PFAS).^80^ Still, some small, polar KUECs, such as 1,4-dioxane and NDMA, can pass through many types of NF and RO membranes (e.g., NDMA removals vary from ∼5–10% with NF to ∼90% with seawater RO),^81,82^ underscoring the value of multibarrier approaches.

Membranes require frequent testing to ensure high-log_10_ pathogen and contaminant removals. While traditional polymeric membranes are structurally strong, they can sometimes exhibit integrity issues that may jeopardize performance. As a safeguard, the integrity of UF membranes is routinely verified to confirm that the membranes or membrane modules have no defects; this involves pressurizing the membranes and measuring the rate of pressure decay to accurately predict log_10_ pathogen removals prior to treatment.^83^ Because high-pressure membranes operate with water recoveries of ∼80–85%, they require ∼15–20% more raw water supply, which may be a problem if water availability is severely limited. NF and RO brine disposal is another challenge we discuss in Residuals Management.

Another concern is fouling, which requires maintenance and can account for ≤20% of a membrane system’s operating cost to restore permeability and prolong lifetime.^84^ Fouling can be categorized into two types: (1) reversible (due to biofilm or particle buildup), with flux restored by backwashing or chemical-enhanced backwash with modest chlorine addition, and (2) irreversible (due to mineral scaling and pore blockage), for which flux is restored by clean-in-place procedures. Antiscalants also can be an effective means of mitigating scaling of NF/RO operations. Although membranes may require chemical addition to mitigate irreversible fouling (and NF and RO usually require remineralization), the cleaning regimes occur offline and do not affect product water quality. In addition, the performance of membrane systems is greatly enhanced when biologically stable water is supplied, which minimizes biofouling and the need for chemical cleaning regimens.^85^ Pretreatment serves as an effective, and often necessary, strategy for combating fouling and extending the life span of membranes by reducing cleaning times, chemical demand, and energy requirements.^86^ Technologies associated with the Minus Approach are effective pretreatments.^87−89^

### 3.4 Improved Water Distribution

After DWT, water is conveyed to customers through DWDS comprised of pipes, pumps, and storage tanks. As water spends more time in DWDS, it is subject to reactions with pipe and tank materials, causing erosion and corrosion. Secondary disinfection minimizes biofilm growth in DWDS, but more disinfectant must be added to prevent microbial recontamination when the detention time is long or when the water contains components that react with the disinfectant.

In the United States, >50% of the population is served water treated with chloramines. Monochloramine is often chosen as the secondary disinfectant because it may offer longer-lasting residuals than free chlorine and produces fewer THMs; however, chloramination comes with a suite of problems. Chloramination can increase lead’s solubility and mobility by reducing Pb(IV) to Pb(II) species; for example, the switch from free chlorine to chloramine was partially responsible for the Washington, DC, lead crisis in the early 2000s.^90^ Also, chloramination produces its own variety of DBPs, including THMs, iodo-DBPs, and nitrosamines,^31^ and can encourage accelerated nitrification and microbial leaching in DWDS, which generates nitrate and nitrite, consumes dissolved oxygen, and increases acidity.^91,92^ For these reasons, Germany disallows the use of chloramines as drinking water disinfectants. In the United States, a disinfection residual is mandated by the Safe Water Drinking Act regulations and is often necessary due to the vast size of DWDS networks (i.e., long hydraulic travel times of up to 2–3 weeks), in which microbial contamination could occur. If DBP precursors can be effectively removed, chlorine or chlorine dioxide is a better alternative than chloramine.

UV disinfection is a rapidly expanding, chemical-free strategy for inactivating pathogens before drinking water enters DWDS or wastewater effluents enter natural water bodies.^93,94^ UV is a powerful disinfection technique that dimerizes base pairs in microbial RNA and DNA to prevent replication and impart a germicidal effect.^95^ New paradigms are emerging for integrating UV at key locations in decentralized water systems, as well as a growing reliance on UV in point-of-use systems within point-of-entry plumbing.^96^ Although it is possible for medium-pressure UV lamps to photolyze nitrate into NO_2_* and OH*, which can spur halonitromethane formation (e.g., chloropicrin) with post-chlorine addition upon reaction with fragmented NOM products,^97^ this poses little problem when nitrate concentrations are low or when low-pressure UV lamps are utilized.

Because many DWDS are aging and experiencing deterioration^98^ and water quality can be adversely affected during distribution,^99^ opportunities for reinvestment should strive to minimize DWDS-based risks. Specifically, a DWDS ideally should have few or no dead ends, short retention times, and proactive leak detection. The use of biostable materials such as stainless steel or polyvinyl chloride can further decrease secondary disinfection requirements,^100^ although this may have significant capital-cost implications. Integrating decentralized treatment processes (e.g., modular UV treatment or other nonchemical technologies) within DWDS is also likely to find a future in the next generation of water systems,^101^ because it provides the added benefits of shorter DWDS detention times and less DBP formation. Operationally, instead of monitoring disinfectant residuals in DWDS for indices of contamination, newer methods for real-time microbial measurements could play a similar role (e.g., online flow cytometry and bacteriological or particle counters).^102,103^

### 3.5 Residuals Management

The benefits of the Minus Approach extend to residuals management. Every separation technology generates some residuals or wastes (e.g., waterborne particles and KUECs removed by adsorbents). The best way to dispose of wastes depends on cost effectiveness and environmental regulations that dictate the extent of treatment necessary prior to disposal. Current residual waste from conventional DWT is dominated by coagulant sludge and spent filter backwash water (SFBW). Coagulant sludge, rich in metals, synthetic coagulant polymers, and organics, is often sent to a landfill or a WWTP or thickened and dried; due to its high water content, sludge transportation and disposal are costly. SFBW is either discharged to sewers or returned to the DWT headworks, the latter of which can result in biological and chemical contamination, including increased levels of DBP precursors, due to accumulation.^104^

The major residuals that the Minus Approach produces are membrane brine and concentrates and SFBW from BF. For the liquid streams, reuse potential can be boosted by reducing overall residual volumes while further concentrating unwanted contaminants. UF is a viable dewatering alternative for SFBW that avoids the drawbacks of recycling to headworks and can recover ≤90% of the volume as a permeate that has a level of pathogens lower than that of raw water.^105^ MF and UF concentrates predominantly consist of removed total suspended solids (TSS), pathogens, turbidity, and cleaning agents and are typically discharged to WWTPs; alternatively, additional membrane stages can further reduce concentrate volumes to varying degrees of purity.^106^

Because NF and RO brines are rich in rejected contaminants and salinity of feedwater, they require more rigorous treatment. Current brine management strategies such as deep disposal wells or evaporation ponds come with large spatial requirements, may induce seismicity, and can contaminate groundwater.^107^ Instead, technologies for zero-liquid discharge (ZLD) can recover significant volumes of clean water from brine while further concentrating waste streams. Various ZLD techniques exist, including membrane methods, electrical methods, and thermal methods;^108^ however, ZLD is constrained by high costs and energy requirements, especially when inland desalination is considered, making it currently not viable to most utilities.^109^ Therefore, the cost-effectiveness of ZLD must be improved through innovative strategies. For example, an inland reuse facility applied BF and novel ion exchange/electrodialysis processes to increase net water yields from 85% to >98% while also addressing management of KUECs, pathogens, and antiscalants in membrane concentrates.^110^ Although chemical-based AOPs (e.g., H_2_O_2_ and UV) have demonstrated themselves as effective technologies for remediating KUECs in concentrated residual streams, newer Minus Approach alternatives such as electrocatalytic methods have been shown to be effective (e.g., defluorination of PFAS-laden wastes) and may become increasingly relevant in the future.^111,112^

### 3.6 Toward more Sustainable Drinking Water

The Minus Approach should create more sustainable DWT, where sustainability includes human health risks, environmental risks, and carbon emissions, because the energy intensities of transportation, purification, storage, distribution, utilization (end use), and disposal of water, energy, water, and carbon are intertwined. In 2015, the annual emissions of greenhouse gases for water utilities in the United States were estimated at 45 million tons.^113^ When considering new infrastructures, life cycle assessments (LCA) and techno-economic assessments (TEA) should guide municipalities toward the most sustainable methods that are economically feasible.

While membranes reduce human health risk due to avoiding DBP formation and providing greater KUEC removals, they usually come with higher capital expenditure (CAPEX) and energy costs (0.05–0.15 and 0.1–0.2 kWh/m^3^ for conventional and UF/MF freshwater treatment, respectively).^114^ Ribera et al. conducted a LCA and human health risk assessment for NF DWT compared to conventional DWT; NF, despite providing an order-of-magnitude reduction in carcinogenic risk (attributed to the reduction of THM formation potential), exhibited higher scores for many negative environmental impact factors due to its higher energy intensity.^115^ A TEA comparing a conventional DWTP to a UF DWTP found that UF, despite requiring 70% less land area and 43% less chemicals, had higher CAPEX, operational expenditure (OPEX) (as energy costs), and overall maintenance costs.^116^ However, continued development (i.e., learning by doing and economies of scale) has improved both CAPEX and OPEX for membranes in recent decades, and similar principles apply to other technologies. From 1977 to 2015, the number of seawater RO plants doubled, while CAPEX decreased by 15%; similar trends have been seen for low-pressure membranes.^117,118^ Technological improvements (e.g., more efficient membrane materials and energy recovery devices) offer further promise to decrease OPEX [e.g., power consumption for RO desalination has drastically decreased in recent decades (>15 kWh/m^3^ in the 1970s to 2.5–4 kWh/m^3^ in 2008)].^119^

Importantly, membrane performance is significantly affected by the mode of operation. Usually, membrane processes are operated at fluxes high enough to compact foulants on the membrane surface, causing significant fouling and necessitating frequent backwashing. This creates higher energy and chemical-cleaning demands, compared to operating at a lower flux, where fouling progresses more slowly.^120^ Thus, achieving an optimal balance of CAPEX with OPEX (i.e., implementing more membrane cassettes that operate at a lower flux instead of fewer cassettes at a higher flux) may improve life cycle cost and performance. Future development in real-time membrane performance monitoring and control should establish energy and cost as primary objectives for optimization.

Renewable energy (RE) technologies, which are rapidly decreasing in price and increasing in availability,^121^ create opportunities to improve the sustainability performance of water treatment. For instance, a LCA comparing NF DWT to conventional DWT modified with GAC showed that when both systems were powered by hydropower, the conventional GAC system had a much worse negative impact factor for human toxicity, climate change, and resource depletion.^122^ Options for additional creativity will involve combining REs and sources of waste energy; a hybrid RO pressure retarded osmosis system utilizing reclaimed wastewater heated by solar thermal energy could reduce desalination energy requirements from 1.1 to 0.39 kWh/m^3^.^123^ REs also offer opportunities to reduce carbon emissions associated with the high energy requirements of UV disinfection and ZLD.^124,125^

Pressurizing and distributing potable water are major energy-based operating costs for public water systems. Approximately 7–8% of the world’s total generated energy is used for drinking water production and distribution,^126^ and many utilities try to leverage off-peak energy demands and prices to limit these costs. While pumped storage in raw water reservoirs is viewed as a strategy for “storing” and utilizing excess energy on daily, weekly, or seasonal domains, it may also be feasible for utilities to explore bidirectional pumps to utilize excess RE when it is available on the grid to pressurize water in DWDS, thus storing energy to use during hourly periods of high demand.^127,128^ Despite the potential of REs, research must address the current technology limitations, and policy factors must harmonize with consumer demand to make suitable selections on a case-by-case basis. Furthermore, as more REs are integrated into DWT, the life cycle trade-offs between energy consumption and the continuous reliance and health effect costs of chlorine-based disinfectants should be more thoroughly evaluated.

## 4 Advancing Water Treatment through Artificial Intelligence

AI and ML offer novel opportunities to enhance water treatment by identifying nonlinear relationships among variables in complex, dynamic systems. Real-time, automated monitoring can fuel models built for water treatment process control and optimization, network monitoring, early anomaly detection, and asset portfolio management, resulting in maximal cost savings.^129^ Two areas where modeling may prove to be particularly advantageous are for source protection and DWDS, the first and last lines of defense. For instance, models have reliably detected point and nonpoint pollution origins in source water and predicted the production of alga-derived substances commonly associated with heightened color and odor instances.^130^ In DWDS, models have efficiently detected pipe bursts for quick rectification of pollution intrusion due to pressure imbalances,^131,132^ segmented large DWDS networks into smaller subsystems that can be more easily managed,^133^ and accurately predicted the sources and timing of microbial contamination.^134^

In addition to preventive maintenance and enhanced prediction power, AI and ML offer great potential for technology optimization. For example, a random forest model reduced cleaning costs by 36% for a UF pilot treating SFBW by adjusting backwashing frequency and duration.^135^ Modeling will also be essential for exploring multifaceted phenomena and providing guidance for designing next-generation technologies. Recently, Bayesian optimization on a ML model built to predict water permeability and salt rejection from various membrane monomer candidates and fabrication conditions allowed for the identification of eight materials that exceeded the present upper bound for water/salt selectivity and permeability.^136^ Importantly, these recommendations are not exclusive to the Minus Approach and can be applied to all areas of water treatment.

## 5 Impediments to the Minus Approach

The Minus Approach relies on source control, multibarrier treatment processes, and alternative DWDS operational schemes. It is impossible to generalize how to best achieve this approach for individual DWT authorities because of the diversity of source water quality, geographical location, land availability, regulatory factors, public acceptance, willingness to pay, and existing infrastructures that make each system unique. Sustainability should drive the future of water management, and the resulting human health, environmental, and economic impacts should be objectively evaluated and considered in unison. Rigorous cost and sustainability analyses are needed to select the most suitable and realistic advancements that can meet the needs of communities based on climate, resource availability, and demand.

It is very likely that water purveyors will need to adopt Minus Approaches to address contemporary challenges, such as the reduction of the levels of DBPs and KUECs based on their discovery and toxicity, for example, treating PFAS beyond perfluorooctanoic acid (PFOA) and perfluorooctanesulfonic acid (PFOS) (e.g., more difficult-to-treat short-chain PFAS and PFAS isomers) to increasingly stringent standards with a reasonable cost. It is clear that Plus Approaches do little to control PFAS,^137^ and individual states have already begun to adopt standards more stringent than the U.S. Environmental Protection Agency’s currently recommended levels of 70 ppt combined PFOA and PFOS.

The sunk costs of existing technologies may create resistance to investment in the Minus Approach. Because the Plus Approach can achieve current potable water standards, it will require financial incentives or more stringent potable water standards to steer a heavily regulated industry toward modern technology. In the United States, small DWT systems account for the majority of drinking water regulatory violations,^138^ many of which are located in marginalized or financially disadvantaged communities. Consolidation of smaller systems into larger systems that have the financial capabilities to meet growing regulatory costs offers the opportunity to install Minus Approach treatment processes.^139^ The authors suggest that goals related to improved water quality, increased public trust, and greater water affordability are clearly stated and described before consolidation takes place. Quantifying the goals for water quality and service outcomes is imperative and is being proposed to the U.S. Environmental Protection Agency as it modifies the Water System Restructuring Rule process.

## 6 Environmental Implications

We introduce the Minus Approach to engage the water community and to ignite interest in designing safer, more sustainable, and more intelligent DWT systems. Because all of its technologies are already available and proven, the Minus Approach can be implemented immediately. Importantly, the Minus Approach is a robust, multibarrier framework, not a prescription or a panacea. Water treatment is a complex sociotechnical topic that depends on source water quality, geography, existing infrastructures, and community culture. Decisions about how to adopt the Minus Approach always will be made on a case-by-case basis, guided by technical, economic, and sustainability factors. Despite local variability, implementing the Minus Approach can redefine the standard of practice of DWT leading to safer drinking water, potentially lower costs, and better sustainability performance.
